# Uterine fibroids with heavy menstrual bleeding stratified by race in a commercial and Medicaid database

**DOI:** 10.1016/j.xagr.2024.100412

**Published:** 2024-10-17

**Authors:** Sanjay K. Agarwal, Michael Stokes, Rong Chen, Cassandra Lickert

**Affiliations:** a Gynecology, and Reproductive Sciences, University of California San Diego, La Jolla, CA (Agarwal); b Evidera, St. Laurent, Quebec, Canada (Stokes); c SimulStat, Solana Beach, CA (Chen); d Sumitomo Pharma America, Inc, Marlborough, MA (Lickert)

**Keywords:** black women, bulk symptoms, hormonal contraceptives, hysterectomy, pharmacotherapy, real-world evidence, surgery/procedures, treatment patterns

## Abstract

**Background:**

Historically, the clinical characteristics and treatment pathways for patients with uterine fibroids and heavy menstrual bleeding have differed between White and Black women.

**Objective:**

To provide a contemporary comparison of patient characteristics and treatment patterns among White and Black women with uterine fibroids and heavy menstrual bleeding in the United States.

**Study Design:**

This retrospective cohort study included administrative claims data from 46,139 White and 17,297 Black women with uterine fibroids and heavy menstrual bleeding from the Optum Clinformatics database (January 2011–December 2020) and 7353 White and 16,776 Black women from the IBM MarketScan Multi-State Medicaid Insurance database (January 2010–December 2019). Patients were indexed at their initial uterine fibroid diagnosis claim and were required to have a claim for heavy menstrual bleeding and ≥12 months of continuous enrollment pre- and postindex. Patients were followed until the earliest of death, disenrollment, hysterectomy date, or end of study database. Outcomes were stratified by race and included patient demographics, clinical characteristics, pharmacologic treatment patterns, and surgeries/procedures. Pearson's Chi-square test for categorical variables and Student's t-test for continuous data were used to evaluate differences in baseline characteristics. Descriptive statistics were used to characterize treatment pathways for hormonal contraceptive use in women with ≥24 months of follow-up. Kaplan–Meier survival analysis was used to estimate time until hysterectomy, with log-rank testing to assess between-group differences.

**Results:**

The mean (standard deviation) duration of follow-up was 44.6 (27.9) and 41.0 (24.9) months in the commercial and Medicaid databases, respectively. Mean (standard deviation) age at uterine fibroid diagnosis was lower for Black than White women in both databases (commercial: 42.3 [6.5] vs 44.4 [6.3] years; *P*<.0001; Medicaid: 39.6 [7.1] years vs 40.2 [7.2] years; *P*<.0001). Anemia was more prevalent in Black vs White women in both databases (commercial: 5.9% [1028/17,297] vs 3.6% [1648/46,139]; *P*<.0001; Medicaid: 7.0% [1180/16,776] vs 4.5% [331/7353]; *P*<.0001). In the commercial database, approximately one-half of women had claims for ≥1 bulk symptom, with no significant differences between groups. In the Medicaid database, significantly more White than Black women had claims for bulk symptoms (77.0% [5665/7353] vs 68.4% [11,477/16,776]; *P*<.0001). Approximately 40% of all patients received hormonal drug therapies as initial treatment, most commonly hormonal contraceptives. However, discontinuation of hormonal contraceptive therapy was nearly universal, with one-half discontinuing within a median treatment duration of ∼5 months. Most women stopped treatment after 1 or 2 agents (commercial: White, 89.9% [9757/10,857]; Black, 90.0% [3594/3993]; Medicaid: White, 92.2% [1635/1773]; Black, 94.2% [4454/4726]). Hysterectomy was the most common procedure, and was more common among White vs Black women (commercial: 43.9% [20,235/46,139] vs 37.8% [6536/17,297]; Medicaid: 46.8% [3444/7353] vs 32.0% [5364/16,776]).

**Conclusions:**

Black women with UF-HMB were diagnosed at a younger age than White women, and White women had higher hysterectomy rates than Black women, representing a shift from earlier researched treatment patterns. Patients with UF-HMB were also highly reliant on hormonal contraceptives, followed by nearly universal therapeutic discontinuation.

**AJOG Global Reports at a Glance**

**Why was this study conducted?**

● Historically, White and Black women with uterine fibroids and heavy menstrual bleeding (UF-HMB) have different clinical characteristics and treatment pathways; contemporary investigation of these patterns is warranted.

**What are the key findings?**

● Black women were diagnosed with UF-HMB at a younger age and had higher rates of anemia than White women.

● White women had higher hysterectomy rates than Black women.

● Hormonal contraceptives were common among both Black and White women; most women discontinued this treatment within 5 months of initiation.

**What does this study add to what is already known?**

● A shift in surgical treatment patterns is underway, with more Black women preferring fertility-sparing treatment.

● Hormonal contraceptive therapy was not an effective treatment for a large proportion of women with UF-HMB.

## Introduction

Uterine fibroids (UF) are benign, hormone-sensitive tumors that occur in women of reproductive age, with incidence increasing until menopause.^1^, ^2^, ^3^, ^4^ In the United States (US), the condition disproportionately affects Black women.^1^, ^2^, ^3^, ^4^, ^5^, ^6^, ^7^, ^8^, ^9^ Once diagnosed, approximately 25% of women with UF have clinically significant symptoms that require treatment.^1^ Based on multiple studies conducted between 2009 and 2012, Black women, compared to White women, experience significantly more frequent and severe UF-associated symptoms. These include heavy menstrual bleeding (HMB), often with associated anemia.^4^ Also common are pelvic pain/pressure, urinary and bowel dysfunction, and leg and lower back pain.^10^, ^11^, ^12^, ^13^, ^14^ No standard-of-care first-line medical therapy is established for UF, and the only definitive treatment is hysterectomy.^1^ Available drugs include hormonal therapies, gonadotropin-releasing hormone (GnRH) analogs, and tranexamic acid.^10^^,^^13^^,^^15^

Research conducted between 2010 and 2015 has shown that up to one-half of US women with symptomatic UF receive pharmacotherapy as initial treatment, with hormonal contraceptive agents being most common after nonsteroidal anti-inflammatory drugs.^16^ In the same study, up to 40% of patients were treated with first-line surgery, most commonly hysterectomy. Historically, hysterectomy rates have been higher among Black than White women.^17^, ^18^, ^19^, ^20^, ^21^ However, more recent evidence suggests changes to this pattern, with Black women preferring uterine-sparing procedures, and avoiding hysterectomy in particular.^4^^,^^5^^,^^22^^,^^23^

Understanding how treatment patterns differ between White and Black women is critical to addressing racial health disparities in UF.^5^ HMB is the most commonly reported symptom in patients with UF,^14^ and a primary driver of patients seeking treatment.^24^ As new medications have recently been approved for women with HMB due to UF, it is important to characterize the existing treatment landscape for these patients.^25^^,^^26^ The objective of this study was to summarize the characteristics and treatment patterns (hormonal and surgical) among commercially insured and Medicaid-enrolled White and Black women with UF-HMB in the US.

## Materials and methods

### Study design

This retrospective cohort study used administrative claims data from the Optum Clinformatics (Optum) and IBM (formerly Truven Health Analytics) MarketScan Multi-State Medicaid Insurance (IBM) databases. Optum data spanned January 1, 2011 to December 31, 2020, and IBM data spanned January 1, 2010 to December 31, 2019 (Supplemental Figure 1). The index date was defined as the first claim corresponding to a UF diagnosis. Follow-up began on the index date and continued until death, disenrollment, hysterectomy, or database end, whichever occurred first.

The Optum database contains longitudinal data from >180 million patients enrolled in primarily commercial health plans. The IBM database contains longitudinal data for >44 million Medicaid enrollees from 12 geographically dispersed states. Both databases contain fully paid and adjudicated claims and provide patient demographics; diagnoses (ICD-9-CM and ICD-10-CM); inpatient/outpatient procedures (Current Procedural Terminology [CPT]-4 and Healthcare Common Procedure Coding System [HCPCS]); and outpatient prescription records (using National Drug Codes [NDCs]). Authors had access to both study databases.

This study was not subject to US Department of Health and Human Services (HHS) Common Rule requirements or Institutional Review Board review because it was conducted using protected health information (PHI), de-identified in compliance with HHS Privacy Rule requirements. All Optum and IBM databases data were obtained from covered entities that permitted this PHI de-identification for research use. Patient consent was not required. Throughout the research process, patient privacy was preserved, and the data owners complied strictly with all applicable Health Insurance Portability and Accountability Act data management rules and the 1964 Helsinki Declaration.

### Study population

Patients had to have a UF diagnosis (1 inpatient claim, or ≥2 outpatient claims ≥30 days apart) during the selection period (Optum: January 1, 2012 to December 31, 2019; IBM: January 1, 2011 to December 31, 2018). Supplemental Table 1 shows corresponding claims codes. Patients were also required to have ≥1 inpatient or outpatient claim with a HMB diagnosis (irrespective of its temporal relationship to the index date); ≥12 months of continuous enrollment prior to and following index (preindex and follow-up periods), or until the date of hysterectomy (for patients who underwent this surgery <12 months following index). Male patients and women <18 or ≥56 years of age on the index date were excluded. This analysis was limited to White or Black women.

### Outcomes

Outcomes were stratified by race (White or Black) and included: demographic characteristics at index; clinical characteristics (identified during the preindex period; Supplemental Table 1) including bulk symptoms; pharmacologic treatment patterns; and surgeries and procedures. The level of comorbidity was assessed using the Charlson Comorbidity Index (CCI).^27^ Pharmacologic treatment patterns were characterized as the number and percentage of patients prescribed drugs for UF-HMB, including hormonal contraceptives, GnRH agonists and antagonists, steroid hormones (androgens and injectable progestin and estrogen derivatives), antifibrinolytics, and analgesics. Treatment pathways were tracked for the patient subgroup treated with hormonal contraceptives with ≥24 months of follow-up, or until hysterectomy for patients who underwent this surgery <24 months following index. Post-index hormonal contraceptive use was tracked for up to 3 agents (ie, up to 2 hormonal contraceptive switches beyond primary therapy). Discontinuation was defined as a gap ≥60 days with no prescription refills. Switching was defined as a new hormonal contraceptive filled prior to discontinuing the previous hormonal contraceptive.

Surgeries/procedures were identified using relevant HCPCS, ICD-9-CM, ICD-10-CM, and/or CPT codes (Supplemental Table 2) and characterized as the number and percentage of patients with surgeries and procedures associated with UF during follow-up. The time from index date to hysterectomy was also evaluated.

### Statistical analysis

Statistically significant differences in baseline characteristics between racial groups were evaluated using Pearson's Chi-square test for categorical variables and Student's t-test for continuous data. Descriptive statistics were used to summarize patient demographics, clinical characteristics, pharmacotherapy use, and surgical procedures. Kaplan–Meier survival analysis was used to identify median time until hysterectomy by race. Log-rank testing was used to assess between-group distributions. All tests of statistical significance were evaluated at the alpha = 0.05 level. Data were analyzed using SAS version 9.4.

## Results

### Demographic and clinical characteristics

A total of 63,436 women with UF-HMB were identified in the commercial database (White 46,139 [72.7%]; Black 17,297 [27.3%]), and 24,129 were identified in the Medicaid database (White 7353 [30.5%]; Black 16,776 [69.5%]; Supplemental Figure 2). The mean (SD [median]) follow-up was 44.6 (27.9 [38.0]) months (3.7 years) in the commercial database and 41.0 (24.9 [35.6]) months (3.4 years) in the Medicaid database. In the commercial database, mean (SD) age at UF diagnosis was lower for Black than White women (42.3 [6.5] years vs 44.4 [6.3] years; *P*<.0001); and more Black than White women (38.9% vs 34.5%; *P*<.0001) were diagnosed with HMB at or after their UF diagnosis (Table 1). Similar patterns were observed in the Medicaid database (White 40.2 [7.2] years vs Black 39.6 [7.1] years; *P*<.0001 and Black 33.0% vs White 26.4%; *P*<.0001, respectively).

**Table 1:** Preindex Demographic Characteristics by Racial Groups

Characteristic	Optum Commercial			IBM Medicaid		
	White (n=46,139)	Black (n=17,297)	*P*-Value^a^	White (n=7353)	Black (n=16,776)	*P*-Value^a^
**Age**			<.0001			<.0001
Mean (SD)	44.4 (6.3)	42.3 (6.5)		40.2 (7.2)	39.6 (7.1)	
Median (range)	45 (41–49)	43 (38–47)		41 (35–46)	40 (35–45)	
**Age group**			<.0001			<.0001
18–29 years	824 (1.8)	542 (3.1)		618 (8.4)	1454 (8.7)	
30–44 years	20,085 (43.5)	9888 (57.2)		4500 (61.2)	10,917 (65.1)	
45–55 years	25,230 (54.7)	6867 (39.7)		2235 (30.4)	4405 (26.3)	
**Geographic location**			<.0001			-
Northeast	6016 (13.0)	1642 (9.5)		-	-	
Midwest	11,895 (25.8)	2563 (14.8)		-	-	
South	18,771 (40.7)	12,104 (70.0)		-	-	
West	9331 (20.2)	919 (5.3)		-	-	
Unknown	126 (0.3)	69 (0.4)		-	-	
**Insurance type**			<.0001			-
Commercial	45,146 (97.8)	16,597 (96.0)		-	-	
Medicare	993 (2.2)	700 (4.0)		-	-	
Medicaid	-	-		7353 (100.0)	16,776 (100.0)	
**HMB diagnosis relative to index (UF diagnosis), n (%)**			<0.0001			<0.0001
>12 months prior	10,356 (22.4)	3512 (20.3)		2194 (29.8)	4619 (27.5)	
>6–12 months prior	3837 (8.3)	1530 (8.8)		631 (8.6)	1476 (8.8)	
0–6 months prior	16,063 (34.8)	5525 (31.9)		2588 (35.2)	5152 (30.7)	
Within 3-months after (inclusive)	7898 (17.1)	3143 (18.2)		1078 (14.7)	2680 (16.0)	
>3 and ≤6 months after	1363 (3.0)	646 (3.7)		185 (2.5)	482 (2.9)	
>6 months after	6622 (14.4)	2941 (17.0)		677 (9.2)	2367 (14.1)	

*EPO*, exclusive provider organization; *HMB*, heavy menstrual bleeding; *HMO*, health maintenance organization; *POS*, point of service; *PPO*, preferred provider organization; *SD*, standard deviation; *UF*, uterine fibroids.

a Comparisons are White vs Black women.

Across both databases, diabetes, hypertension, and obesity were more common among Black women (Table 2). Among White women, fatigue, female genital cancers, depression, and anxiety were more common. Anemia was prevalent in 3.6% and 4.5% of White and 5.9% and 7.0% of Black women in the commercial and Medicaid databases, respectfully (*P*<.0001 for both). In the commercial database, there was no significant difference in bulk symptom frequency between Black and White women (49.6% vs 49.8%), although significant differences did exist for individual symptoms (Table 2, Supplemental Figure 3). Bulk symptoms that were more common among Black women were pelvic pain/pressure and leg pain, while White women had higher rates of backache and urinary frequency/incontinence. In the Medicaid database, significantly more White than Black women (77.0% vs 68.4%; *P*<.0001) experienced bulk symptoms; this pattern existed for all individual symptoms except constipation, which occurred with equal frequency across races.

**Table 2:** Preindex Clinical Characteristics by Racial Groups

Characteristic	Optum Commercial			IBM Medicaid		
	White (n=46,139)	Black (n=17,297)	*P*-Value^a^	White (n=7353)	Black (n=16,776)	*P*-Value^a^
**CCI score, n (%)**			<.0001			<.0001
0	35,518 (77.0)	12,968 (75.0)		4026 (54.8)	9810 (58.5)	
1	6999 (15.2)	2845 (16.4)		1962 (26.7)	4052 (24.2)	
2	2274 (4.9)	759 (4.4)		691 (9.4)	1279 (7.6)	
3+	1348 (2.9)	725 (4.2)		674 (9.2)	1635 (9.7)	
**CCI score, mean (SD)**	0.4 (0.9)	0.4 (1.1)	<.0001	0.8 (1.4)	0.8 (1.5)	NS
**Most common individual CCI comorbidities, n (%)**						
Chronic pulmonary disease	4700 (10.2)	1783 (10.3)	NS	2006 (27.3)	3697 (22.0)	<.0001
Diabetes without chronic complication	2596 (5.6)	1589 (9.2)	<.0001	1107 (15.1)	2736 (16.3)	.0143
Any malignancy, including lymphoma and leukemia, except malignant neoplasm of skin	1594 (3.5)	354 (2.0)	<.0001	232 (3.2)	386 (2.3)	.0001
**Comorbidities related to women's health, n (%)**						
Infertility	1097 (2.4)	451 (2.6)	NS	35 (0.5)	135 (0.8)	.0050
Pregnancy	1948 (4.2)	927 (5.4)	<.0001	528 (7.2)	1385 (8.3)	.0044
Uterine rupture	1 (0.0)	0 (0.0)	NS	0 (0.0)	0 (0.0)	NS
Endometriosis	2486 (5.4)	707 (4.1)	<.0001	318 (4.3)	322 (1.9)	<.0001
Uterine polyps	2123 (4.6)	500 (2.9)	<.0001	242 (3.3)	310 (1.8)	<.0001
Adenomyosis	501 (1.1)	164 (0.9)	NS	137 (1.9)	153 (0.9)	<.0001
**Bulk symptoms, n (%)**						
Bulk symptoms	22,983 (49.8)	8586 (49.6)	NS	5665 (77.0)	11,477 (68.4)	<.0001
Abdominal distention	1171 (2.5)	328 (1.9)	<.0001	222 (3.0)	336 (2.0)	<.0001
Backache	8262 (17.9)	2886 (16.7)	.0003	2894 (39.4)	4816 (28.7)	<.0001
Constipation	1829 (4.0)	894 (5.2)	<.0001	730 (9.9)	1644 (9.8)	NS
Increased abdominal girth	1826 (4.0)	653 (3.8)	NS	339 (4.6)	665 (4.0)	.0207
Leg pain	4537 (9.8)	1813 (10.5)	.0154	1624 (22.1)	3129 (18.7)	<.0001
Pelvic pressure/pain	12,418 (26.9)	4871 (28.2)	.0017	3990 (54.3)	7916 (47.2)	<.0001
Urinary frequency/incontinence	4647 (10.1)	1591 (9.2)	.0010	1427 (19.4)	2416 (14.4)	<.0001
**Mental health disorder, n (%)**						
Anxiety	6920 (15.0)	1588 (9.2)	<.0001	2993 (40.7)	2976 (17.7)	<.0001
Depression	5795 (12.6)	1415 (8.2)	<.0001	2802 (38.1)	3898 (23.2)	<.0001
**Gastrointestinal conditions, n (%)**						
Diarrhea	2013 (4.4)	629 (3.6)	<.0001	795 (10.8)	1019 (6.1)	<.0001
Nausea/vomiting	3350 (7.3)	1451 (8.4)	<.0001	1785 (24.3)	3057 (18.2)	<.0001
**Other conditions, n (%)**						
Hypertension	8824 (19.1)	5309 (30.7)	<.0001	2398 (32.6)	7389 (44.0)	<.0001
Hyperlipidemia	8496 (18.4)	3098 (17.9)	NS	1794 (24.4)	3196 (19.1)	<.0001
Urinary tract infection	5912 (12.8)	2384 (13.8)	.0013	1840 (25.0)	3717 (22.2)	<.0001
Fatigue	8664 (18.8)	2774 (16.0)	<.0001	1748 (23.8)	2844 (17.0)	<.0001
Anemia	1648 (3.6)	1028 (5.9)	<.0001	331 (4.5)	1180 (7.0)	<.0001
Obesity	5538 (12.0)	3101 (17.9)	<.0001	1886 (25.6)	4841 (28.9)	<.0001

*CCI*, Charlson Comorbidity Index; *NS*, not significant; *SD*, standard deviation.

a Comparisons are White vs Black women.

### Pharmacologic treatment patterns

**Figure 1:**
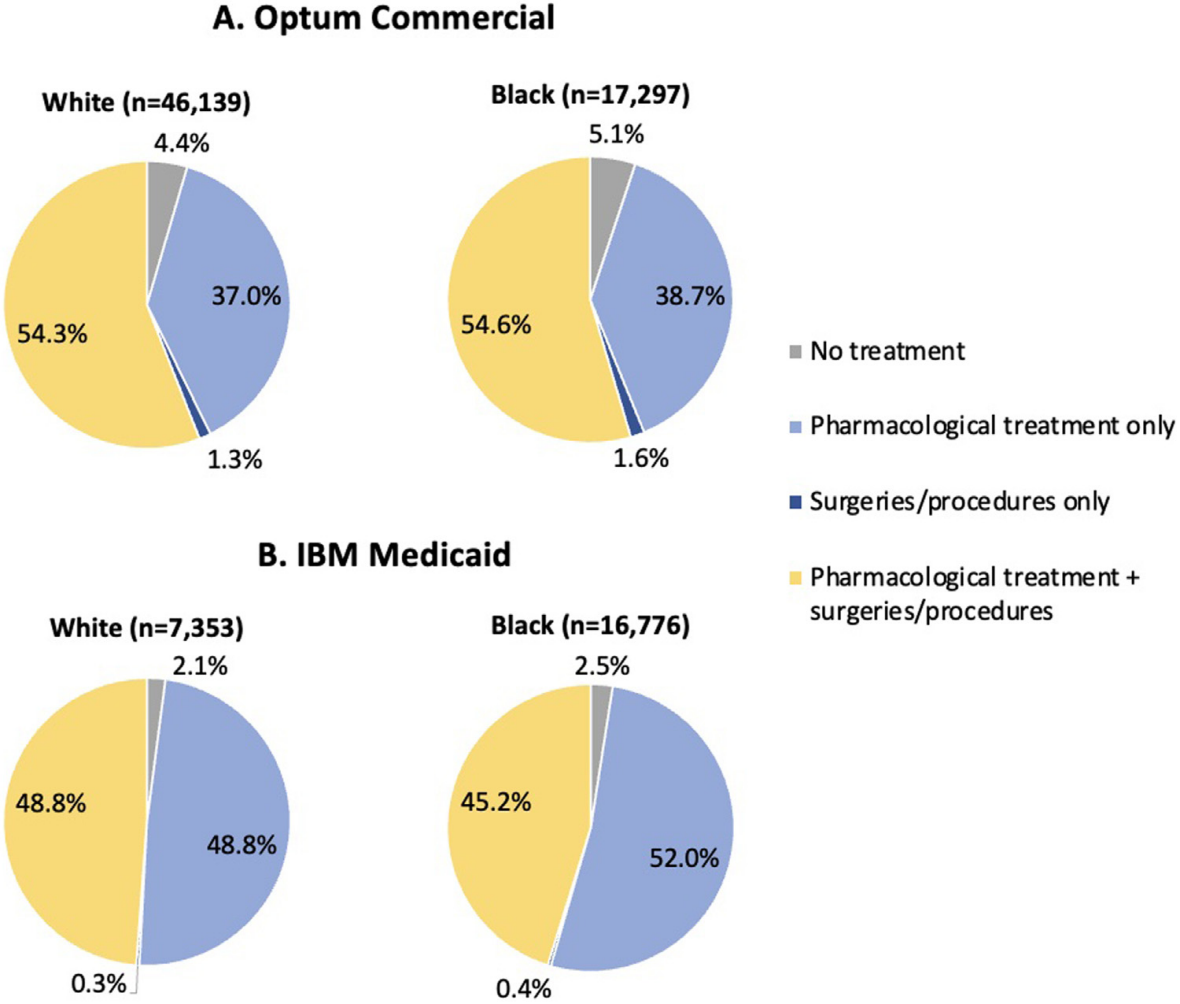
Overall Treatment Patterns, Use of Surgeries/Procedures and Pharmacotherapy During Follow-Up: (A) Optum Commercial and (B) IBM Medicaid Databases

Figure 1 shows overall postindex treatment patterns. In the commercial database, 54.3% of White and 54.6% of Black women received pharmacologic treatment plus surgeries/procedures, while 37.0% of White and 38.7% of Black women received pharmacologic treatment only. In the Medicaid database, 48.8% of White and 45.2% of Black women received pharmacologic treatment plus surgeries/procedures, while 48.8% of White and 52.0% of Black women received pharmacologic treatment only.

Agarwal. Uterine fibroids with heavy menstrual bleeding stratified by race in a commercial and Medicaid database. Am J Obstet Gynecol MFM 2024.

Across both databases and races, 35.3% to 42.4% of women received hormonal treatment as initial therapy (Supplemental Table 3). Most common were hormonal contraceptives alone (commercial: White 64.2%, Black 64.5%; Medicaid: White 57.7%, Black 55.2%), followed by hormonal contraceptives plus steroid hormones (Commercial: White 19.4%, Black 26.5%; Medicaid: White 34.4%, Black 38.0%), and steroid hormones alone (commercial: White 15.6%, Black 7.8%; Medicaid: White 4.7%, Black 2.2%). The mean (SD) duration of contraceptive use was longer for Black than White women (377.3 [522.2] vs 323.1 [460.2] days; *P*<.001). Very few women (<5%) were treated with GnRH agonists or antagonists, either initially or during follow-up.

**Figure 2:**
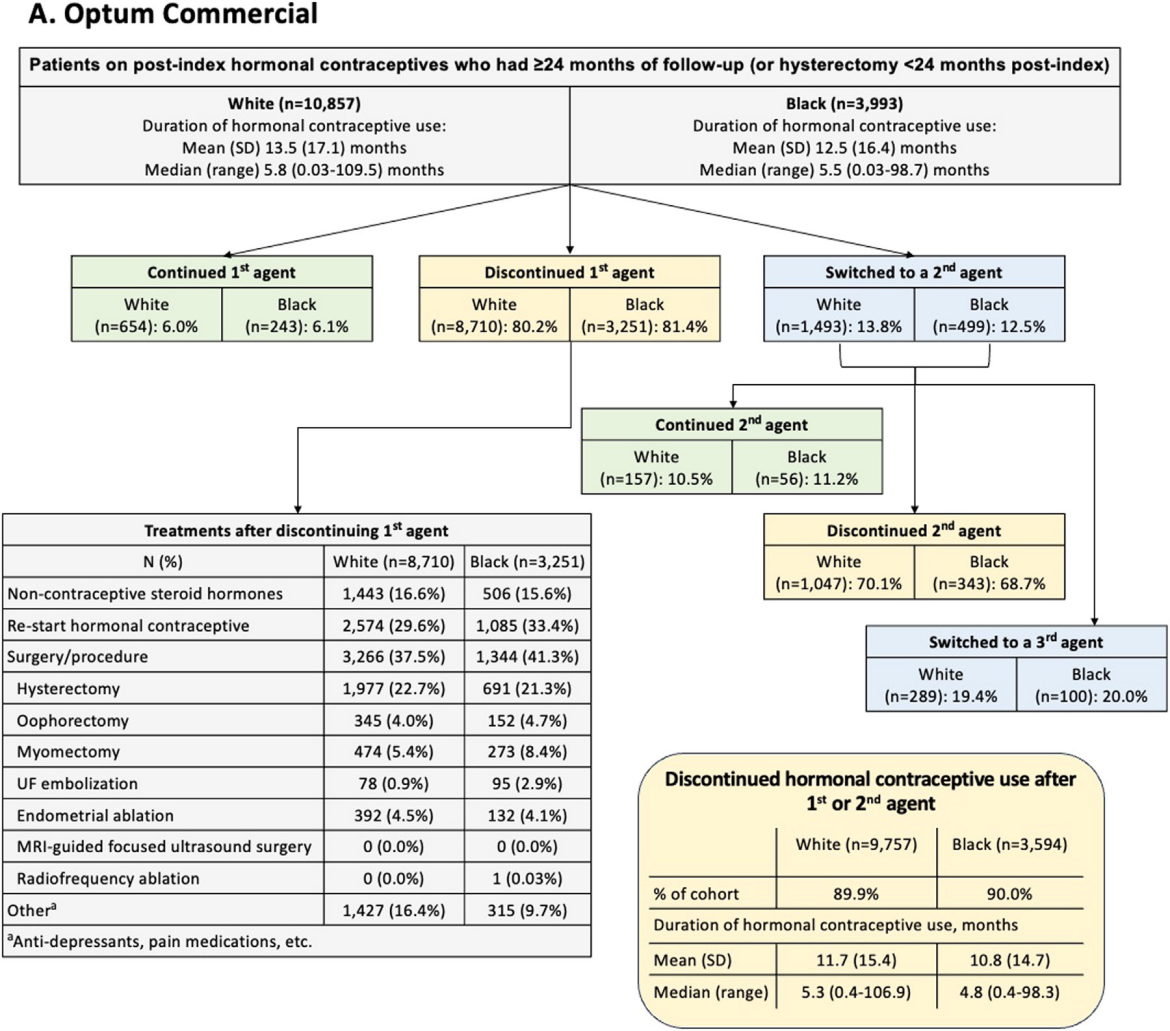

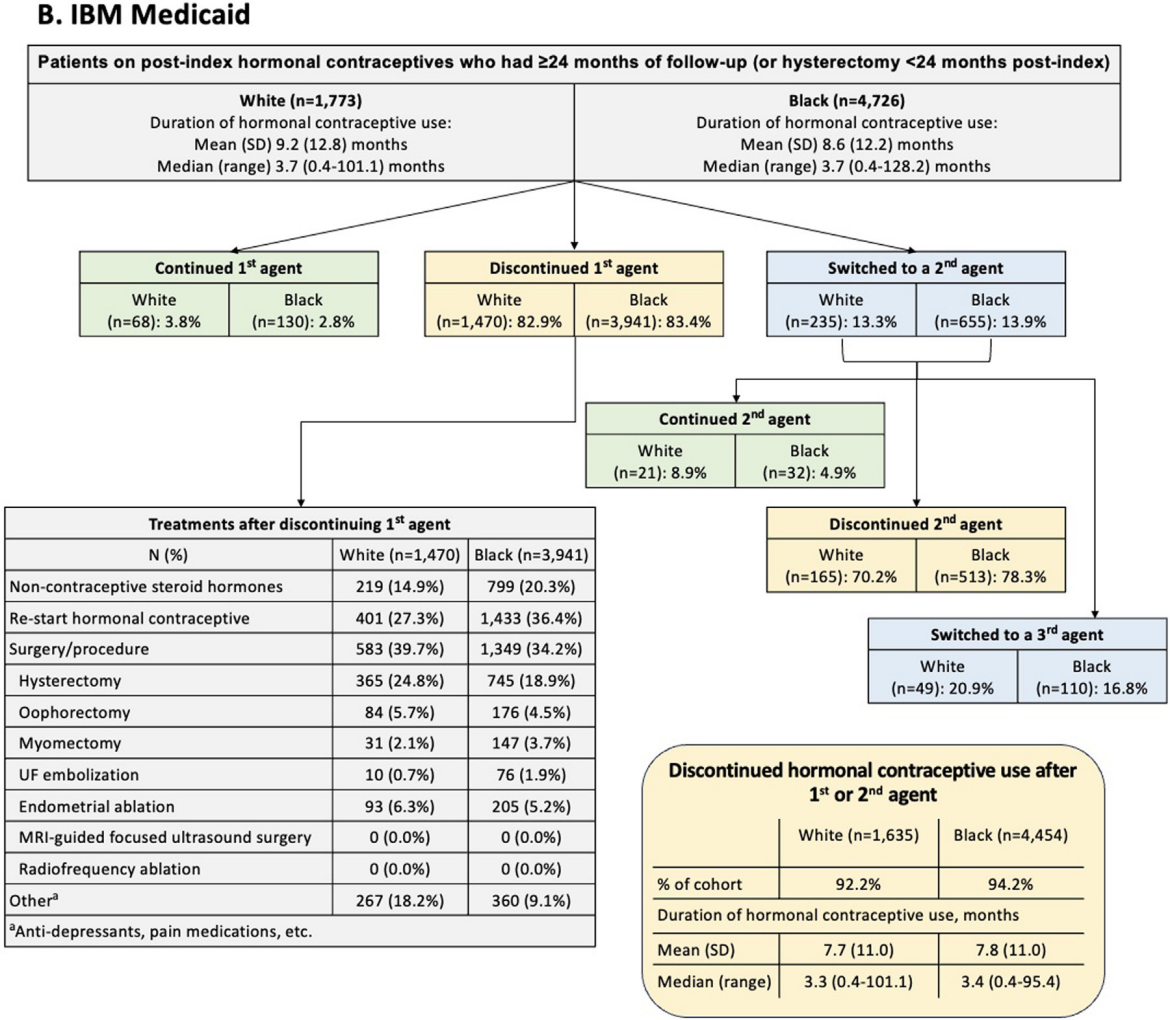
Post-Index Treatment Pathway for Hormonal Contraceptive Agents, (A) Optum Commercial and (B) IBM Medicaid Abbreviations: *MRI*, magnetic resonance imaging; *UF*, uterine fibroid.

Figure 2 summarizes data for women who used hormonal contraceptives and had ≥24 months of follow-up or hysterectomy <24 months post-index. This subgroup comprised approximately one-quarter of the overall dataset (commercial: White n=10,857, Black n=3993; Medicaid: White n=1773, Black n=4726). After initiating a first hormonal contraceptive, fewer than ∼15% of patients switched to a second hormonal contraceptive (commercial: White 13.8%, Black 12.5%; Medicaid: White 13.3%, Black 13.9%). Of the women who initiated a second hormonal contraceptive, approximately one-fifth switched to a third hormonal contraceptive (commercial: White 19.4%, Black 20.0%; Medicaid: White 20.9%, Black 16.8%). However, rather than switching, most women discontinued hormonal contraceptive use after 1 or 2 agents (commercial: White 89.9%, Black 90.0%; Medicaid: White 92.2%, Black 94.2%). These discontinuations occurred following a median treatment duration of ∼5 months for commercially insured patients (White 5.3 months, Black 4.8 months) and ∼3 months for Medicaid patients (White 3.3 months, Black 3.4 months). After discontinuing initial hormonal contraceptive treatment, many women went on to have a surgery or procedure (commercial: White 37.5%, Black 41.3%; Medicaid: White 39.7%, Black 34.2%), with hysterectomy being the most common (commercial: White 22.7%, Black 21.3%; Medicaid: White 24.8%, Black 18.9%). The exception was Medicaid-insured Black women, who most commonly went on to reinitiate hormonal contraceptive therapy after a gap of ≥60 days (commercial: White 29.6%, Black 33.4%; Medicaid: White 27.3%, Black 36.4%).

Agarwal. Uterine fibroids with heavy menstrual bleeding stratified by race in a commercial and Medicaid database. Am J Obstet Gynecol MFM 2024.

### Surgeries and procedures

**Figure 3:**
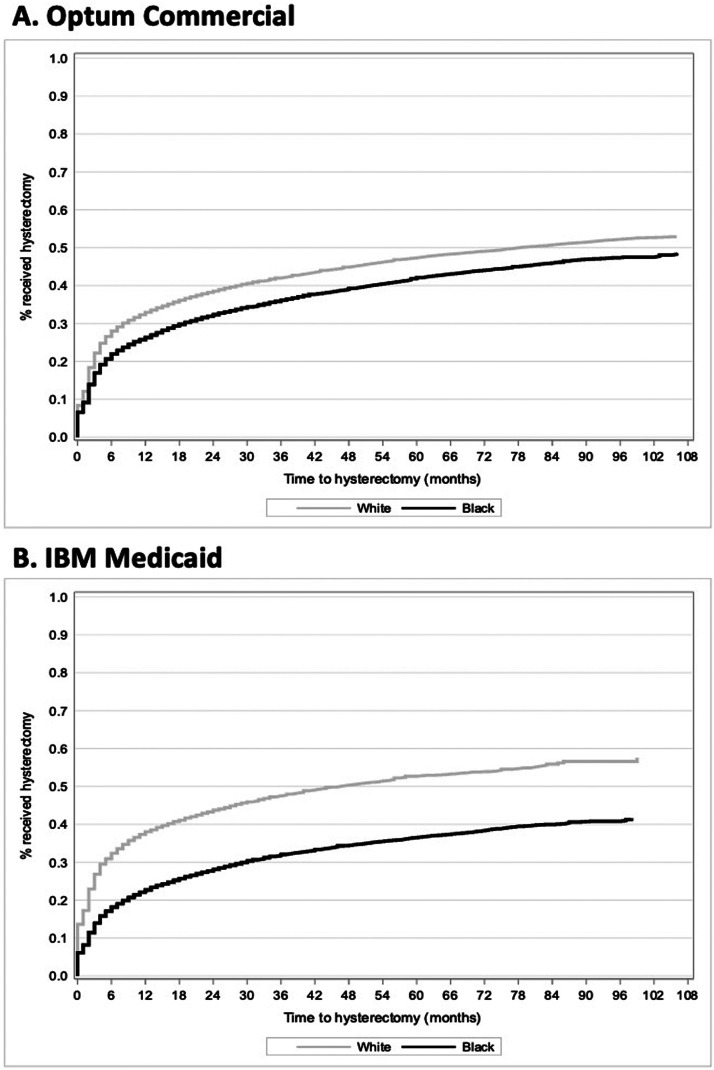
Median Time To Hysterectomy in Commercial White (A), Black (B), and Medicaid White (C) and Black (D) Cohorts.

Across both databases and races, hysterectomy was the most common surgery/procedure during the follow-up period (commercial: White 43.9%, Black 37.8%; Medicaid: White 46.8%, Black 32.0% [Table 3]). Approximately 5% of women underwent ≥2 surgical procedures, with a median (range) time from first to second surgery of 10 (4–23) months to 12 (4–27) months. Figure 3 shows KM analyses of time to hysterectomy. At 12 and 24 months, respectively, the following percentages of women had received a hysterectomy: Commercial, White 32.9% and 38.5%, Black 26.3% and 32.4%; Medicaid, White 38.0% and 43.8%, Black 22.7% and 28.1%.

**Table 3:** Surgeries/Procedures of Interest During Follow-Up by Race

Characteristic	Optum Commercial		IBM Medicaid	
	White (n=46,139)	Black (n=17,297)	White (n=7353)	Black (n=16,776)
**Surgeries/procedures, n (%)**				
Hysterectomy	20,235 (43.9)	6536 (37.8)	3444 (46.8)	5364 (32.0)
Radical hysterectomy	332 (0.7)	98 (0.6)	70 (1.0)	103 (0.6)
Hysterectomy with oophorectomy	32 (0.1)	12 (0.1)	3 (0.0)	3 (0.0)
Total hysterectomy alone	17,652 (38.3)	5580 (32.3)	3071 (41.8)	4,532 (27.0)
Supra-cervical hysterectomy alone	2075 (4.5)	782 (4.5)	169 (2.3)	495 (3.0)
Other	144 (0.3)	64 (0.4)	131 (1.8)	231 (1.4)
Oophorectomy	4,865 (10.5)	1693 (9.8)	1109 (15.1)	1463 (8.7)
Myomectomy	4,514 (9.8)	2243 (13.0)	262 (3.6)	982 (5.9)
UF embolization	756 (1.6)	687 (4.0)	84 (1.1)	492 (2.9)
Endometrial ablation	3798 (8.2)	1088 (6.3)	729 (9.9)	1274 (7.6)
MRI-guided focused ultrasound surgery	4 (0.0)	1 (0.0)	0 (0.0)	0 (0.0)
Radiofrequency ablation (laparoscopic, transvaginal, or transcervical)	10 (0.0)	6 (0.0)	0 (0.0)	1 (0.0)
Laparoscopic	10 (0.0)	6 (0.0)	0 (0.0)	1 (0.0)
Transcervical	0 (0.0)	0 (0.0)	0 (0.0)	0 (0.0)
**Patients with ≥2 surgical procedures, n (%)**	2347 (5.1)	786 (4.5)	399 (5.4)	682 (4.1)
**Time (in months) from first to second surgery**				
Mean (SD)	16.9 (18.3)	19.4 (20.5)	15.3 (13.6)	17.1 (16.6)
Median (range)	10 (4–23)	12 (4–27)	11 (5–22)	12 (5–23)

*MRI*, magnetic resonance imaging; SD, standard deviation; UF, uterine fibroid.

Agarwal. Uterine fibroids with heavy menstrual bleeding stratified by race in a commercial and Medicaid database. Am J Obstet Gynecol MFM 2024.

## Comment

### Principal findings

At UF diagnosis, Black women were on average 1 to 2 years younger than White women. UF disease burden was substantial for all patients; approximately 50% to 80% of Medicaid patients reported bulk symptoms, with a significantly higher rate among White (77%) than Black (68%) women. In the commercial database, both races had similar bulk symptom rates (50%). Only a small proportion of patients (4%–7%) had documented anemia, which was significantly more common among Black women.

In both databases, approximately 35% to 42% of patients received hormonal drug therapy as initial treatment, most commonly hormonal contraceptives. However, hormonal contraceptive therapy discontinuation was nearly universal, with 90% to 94% of women stopping after 1 or 2 agents, and one-half discontinuing within ∼5 months of initiation. Fewer than 15% of women received a uterine-sparing procedure. Hysterectomy was the most common procedure and more common in White women (44% commercial, 47% Medicaid) than Black women (38% commercial, 32% Medicaid). Within 1 year of diagnosis, approximately one-third of White women and one-quarter of Black women had a hysterectomy.

### Results in the context of what is known

In this analysis, Black women were diagnosed at a younger age than White women, and Medicaid-insured White women had a higher symptom burden than Black women. Although differences in prior study designs make it difficult to interpret findings and assertions,^2^^,^^4^^,^^5^^,^^23^^,^^28^ research has shown that Black women with UF are diagnosed at a younger age, are more symptomatic, and have more severe symptoms than White women.^2^^,^^4^^,^^5^^,^^23^ In a prospective national survey analysis (2001–2002; N=1364) by Baird et al.,^2^ premenopausal Black and white women were diagnosed at a mean 33 vs 36 years (*P*<.001). Additionally, the 2015 Comparing Options for Management: Patient-Centered Results for UFs (COMPARE-UF) treatment registry found that Black women had significantly higher rates of pelvic pain (56.3%) than White women (46.8% *P*<.05).^23^

The current study showed lower rates of anemia (4%–7%), a hallmark of UF and HMB,^10^, ^11^, ^12^, ^13^, ^14^ than previous analyses suggesting that 20% to 40% of UF-HMB patients have HMB-induced anemia.^5^ Given that all study participants had a HMB claim, this low prevalence likely represents underreporting. Indeed, the current study relied solely on ICD codes to identify anemia, whereas previous analyses used laboratory-measured hemoglobin concentrations.^5^ Despite this, consistent with historical data, Black women were more likely than White women to have anemia.^4^^,^^5^ In a national web-based survey of women with UF (N=841), Stewart et al.,^4^ found that Black women had significantly higher rates of anemia than White women (adjusted risk ratio: 2.73 95% CI [1.47, 5.09]). The higher anemia rate among Black women in the current analysis might be related to a temporal pattern observed with respect to HMB and UF diagnosis, wherein Black women were more commonly diagnosed with HMB at or after their UF diagnosis. In contrast, White women were more commonly diagnosed with HMB prior to UF, which may have led to earlier intervention for and resolution of anemia.

Consistent with prior research, this study confirmed that hormonal contraceptive therapies are widely used to treat UF, and frequently discontinued. A 2010 to 2015 treatment utilization claims analysis of US women with symptomatic UF (Bonine et al.,^16^) found that hormonal contraceptives comprised 39.6% and 20.1% of initial pharmacotherapy among commercially and Medicaid-insured women. Researchers did not analyze treatment pathways following hormonal contraceptive use, but 40.5% to 47.5% of women who initiated any pharmacotherapy received a second treatment, implying that initial treatment was inadequate for nearly one-half of women.

The current study also supports historical research showing that hysterectomy rates remain high for all women with UF, regardless of race.^5^^,^^23^^,^^28^ Importantly, this study shows higher hysterectomy rates for White than Black women, which represents a notable shift from research conducted before 2010.^18^^,^^19^ The population-based National Hospital Discharge survey (1988–1990) showed that Black women received hysterectomy for UF at double the rate of White women (37.6 vs 16.4 per 10,000).^19^ A 2007 study evaluating UF hospitalizations using data from the Nationwide Inpatient Sample (N=355,135) found that Black women had hysterectomy at a rate 2.4 times that of White women.^18^ However, more recent research, including COMPARE UF, has shown a trend towards the increased use of uterine-sparing procedures in Black women, favoring myomectomy and uterine artery embolization over hysterectomy.^23^ Likewise, a 2010 to 2018 retrospective cohort analysis of US Veterans (Katon et al.,^5^) with newly-diagnosed UF (N=8247) found that Black women were less likely than White women to have a hysterectomy as initial UF treatment, and more likely to select a uterine-sparing procedure. Whether these shifts in hysterectomy rates among Black women reflect a change in preferences and/or structural factors (eg, access, cost) cannot be ascertained from claims data. However, these data should be interpreted within the context of historical injustices imposed on Black women that may have affected access to uterine-sparing procedures, and that may engender persistent mistrust towards healthcare providers*.*^9^^,^^29^

## Clinical implications

The observed disparity in claims for anemia between Black and White women with UF, along with the delay in HMB diagnosis for Black vs White women, warrants increased vigilance on behalf of clinicians for the presence of HMB and anemia among Black women.

This study also points to the underutilization of available uterine-sparing treatments, with <15% of women undergoing oophorectomy or myomectomy, <4% undergoing UF embolization, and <1% undergoing MRI-guided focused ultrasound surgery or radiofrequency ablation. The causes of this low utilization, for example, cost concerns, patient awareness and/or physician training, UF severity, and/or concerns about clinical efficacy, requires further investigation. Regardless, patients should be engaged in shared decision-making^1^ and made aware of the benefits and risks of these options, especially patients desiring future pregnancy.

Finally, this study identifies an ongoing need for more effective oral treatment for women with UF. The high rate of hormonal contraceptive therapy discontinuation indicates that these medications are not adequate in terms of efficacy or tolerability for a large proportion of women. Furthermore, few patients (<5%) utilized GnRH agonist/antagonist therapy. This is not surprising because, prior to 2020, GnRH antagonists were not approved and agonists were recommended primarily as preoperative bridging for surgical intervention, due to adverse effects.^30^ Since this study was conducted, 2 new oral GnRH antagonists with add-back hormonal therapy, elagolix and relugolix, have been approved for HMB associated with UF.^25^^,^^26^ The add-back therapy offsets the hypoestrogenic effects of GnRH antagonism, making them safe for up to 24 months of use.^1^^,^^25^^,^^26^ In their 2021 UF guidelines, the American College of Obstetricians and Gynecologists recommend GnRH antagonists with add-back therapy, especially for women with UF seeking to preserve fertility.^1^

## Research implications

Patients had extremely high rates of hormone therapy discontinuation, regardless of race or database, highlighting a need to investigate the rationale behind such treatment choices. In addition, future treatment patterns may change based on updated guidance.^1^ Additional research will be useful to observe these shifts in treatment uptake and retention, both overall and across races.

Further research is warranted to better characterize and understand the drivers of treatment patterns among Black and White women with UF. It is widely accepted that the racial health disparities evident in UF result from bias due to racism and the impact of other social determinants of health, although the ways in which these mechanisms manifest as inequitable care require further elucidation.^1^^,^^5^ Despite being more affected by UF,^1^^,^^6^ Black women have been documented to comprise only 15% of participants in UF studies worldwide.^31^ Future research should, therefore, be careful to avoid underrepresentation of black women.

## Strengths and limitations

This research helps to address the historic under-reporting of research outcomes in US Black women by evaluating both commercial and Medicaid patients drawn from a large, representative population. Black women represented the majority (69.5%) of participants in the Medicaid database, and 27.2% of the commercial database.

Several limitations should be noted. This analysis provides no insight into factors contributing to physician decision-making, nor is it known whether patients were treated with their preferred therapy. Whether patients had UF diagnoses or uterine surgeries prior to the study period is unknown and may have impacted treatment choices during the study. Although medication claims indicated prescribing patterns, it is not possible to confirm the medication was consumed. It is also possible that some medications used, such as contraceptive hormone therapy, were prescribed and/or discontinued for other purposes. Medical records were not accessed to corroborate these claims data. Additionally, since research was not the primary purpose of these electronic health record datasets, administrative ICD-9/10 diagnosis and CPT codes could be inaccurate or included as “rule-out” diagnoses. However, all diagnosis and procedure codes were compared to those in effect when raw data were collected, and edited if necessary. To minimize patient misclassification, UF cases were required to have ≥2 outpatient claims ≥30 days apart or 1 inpatient claim with a UF diagnostic code. Finally, this study sample may not be generalizable to older patients.

## Conclusion

In this large, retrospective claims analysis, Black women with UF-HMB were diagnosed at a younger age than White women, and White women had higher hysterectomy rates than Black women, representing a shift from earlier research treatment patterns. Hormonal contraceptive use was common, although discontinuation within a short timeframe was nearly universal.

## CRediT authorship contribution statement

**Sanjay K. Agarwal:** Writing – review & editing, Visualization, Supervision, Methodology. **Michael Stokes:** Writing – review & editing, Visualization, Validation, Supervision, Methodology, Investigation, Formal analysis, Data curation. **Rong Chen:** Software, Formal analysis, Data curation. **Cassandra Lickert:** Writing – review & editing, Writing – original draft, Supervision, Project administration, Methodology, Formal analysis, Conceptualization.

## Appendix Supplementary materials

Image, application 1

## Footnotes

Supplementary material associated with this article can be found in the online version at doi:10.1016/j.xagr.2024.100412.
