# Supplementary material for: Uterine fibroids with heavy menstrual bleeding stratified by race in a commercial and Medicaid database
Source: AJOG Glob Rep. 2024 Oct 17;4(4):100412. doi: 10.1016/j.xagr.2024.100412 (PMC11570315; doi:10.1016/j.xagr.2024.100412)
Supplement: Supplementary file 1 [file mmc1.docx]

**Supplementary Appendix**

Supplemental **Table 1. ICD-9 and ICD-10 Codes and Definitions for Comorbidities/Conditions Related to Women’s Health, Bulk Symptoms, and Other Comorbidities**

|  | **ICD-9** | **ICD-10** |
| --- | --- | --- |
| **Comorbidities/conditions related to women’s health** | | |
| **Adenomyosis** | 617.0 | N80.0 |
| **Endometriosis** | 617.0, 617.1-617.6, 617.8, 617.9, 622.2 | N80.0-N80.6, N80.8. N80.9 |
| **Infertility** | 628.0-628.4, 628.8, 628.9, 867.4, 878.4, 878.6, 922.4 | N97.0 -N97.4, N97.8, N97.9, E23.0 |
| **Pregnancy** | V22, V23, 650, 651 | Z33.1, Z34, Z3A |
| **Uterine polyps** | 621.0, 622.7, 623.7, 624.6 | N84.0 -N84.3, N84.8, N84.9 |
| **Uterine rupture** | 665.00, 665.01, 665.03, 665.10, 665.11 | O71.00, O71.02, O71.03, O71.1 |
| **Bulk symptoms** | **ICD-9** | **ICD-10** |
| **Abdominal distention** | 787.3 | R14.0, R14.1, R14.2, R14.3 |
| **Backache** | 724.1-724.5 | M54.14-M54.18, M54.30-M54.32, M54.40-M54.42, M54.5, M54.6, M54.89, M54.9 |
| **Constipation** | 564.0 | K59.0 |
| **Increased abdominal girth** | 789.30-789.37, 789.39 | R19.00-R19.07, R19.09 |
| **Leg pain** | 729.5 | M79.604-M79.606, M79.609, M79.651, M79.652, M79.659, M79.661, M79.662, M79.669  M79.671-M79.676 |
| **Pelvic pressure/pain** | 625.0, 625.3, 789.00, 789.03, 789.04, 789.07, 789.09, 789.63, 789.64, 789.9 | R10.84, R10.9, R19.8, N94.1,  N94.4-N94.6, R10.30-R10.32, R10.813, R10.814, R10.823, R10.824 |
| **Urinary frequency/**  **incontinence** | 788.1, 788.20, 788.21, 788.29, 788.3, 788.63, 788.41 | R30.0, R30.9, R32, R33.0, R33.8, R33.9, R35.0, R39.14, R39,15 |
| **Other comorbidities** | **ICD-9** | **ICD-10** |
| **Anemia** | 280.0, 285.1 | D50.0, D62 |
| **Anxiety** | 300.0, 300.2, 300.3, 09.81 | F43.22, F43.23, F40-F42, F43.0, F43.1, F43.8, F43.9 |
| **Chronic pulmonary disease** | 416.8, 416.9, 490.x-505.x, 506.4, 508.1, 508.8 | I27.8, I27.9, J40.x-J47.x, J60.x-J67.x, J68.4, J70.1, J70.3 |
| **Depression** | 296.1-296.3, 296.5, 300.4, 309.0, 311 | F32, F33.0-F33.3, F33.8, F33.9, F34.1, F41.2, F43.21, F31.30-F31.32, F31.4, F31.5, F31.75, F31.76 |
| **Diabetes without chronic complications** | 250.0-250.3, 250.8, 250.9 | E10.0, E10.1, E10.6, E10.8, E10.9, E11.0, E11.1, E11.6, E11.8, E11.9, E12.0, E12.1, E12.6, E12.8, E12.9, E13.0, E13.1, E13.6, E13.8, E13.9 |
| **Diabetes with chronic complications** | 250.4-250.7 | E10.2-E10.5, E10.7, E11.2-E11.5, E11.7, E12.2-E12.5, E12.7, E13.2-E13.5, E13.7 |
| **Fatigue** | 780.79, 780.71 | G93.3, O26.811-O26.813, O26.819, R53.0, R53.82, R53.83 |
| **Hyperlipidemia** | 272.0-272.4 | E78.0-E78.5 |
| **Hypertension** | 401.0, 401.1, 401.9, 402.0, 402.1, 402.9, 403.0, 403.1, 403.9, 404.0, 404.1, 404.9, 405.0, 405.1, 405.9 | I10, I11.0, I11.9, I12.0, I12.9, I13.0-I13.2, I13.9, I15.0-I15.2, I15.8, I15.9 |
| **Nausea/vomiting** | 787.0, 078.82, 536.2, 569.87 | R11 |
| **Obesity** | 278.0 | E66.x |
| **Urinary tract infection** | 590, 595, 597, 598, 599.1-599.5, 599.69, 599.0, 996.64 | A56.01, N10, N11.0, N11.1, N11.8, N11.9, N12, N13.6, N13.9, N15.1, N15.9, N16, N28.84-N28.86, N30.0, N30.10, N30.11, N30.20, N30.21, N30.30, N30.31, N30.40, N30.41, N30.80, N30.81, N30.9, N30.90, N30.91, N34.0-N34.3, N35.011-N35.013, N35.021, N35.028, N35.12, N35.8, N35.9, N36.0, N36.1, N36.5, N36.8, N37, N39.0, N99.115, N99.12, T83.510A, T83.511A, T83.512A, T83.518A |

Abbreviation: ICD = International Classification of Diseases.

Supplemental **Table 2. Surgery and Procedure Codes**

| **Surgery or procedure** | **CPT/HCPCS** | **ICD-9-CM** | **ICD-10-PCS** |
| --- | --- | --- | --- |
| **Endometrial ablation** | 58353, 58356, 58563, 56356, 0071T, 0072T, 49203, 49204, 49205 | 68.23 | 0U5B0ZZ, 0U5B3ZZ, 0U5B4ZZ, 0U5B7ZZ, 0U5B8ZZ, 0UDB7ZZ, 0UDB8ZZ |
| **Hysterectomy** | 58150, 58152, 58180, 58200, 58210, 58240, 58260, 58262, 58263, 58267, 58270, 58275, 58280, 58285, 58290, 58291, 58292, 58293, 58294, 58541, 58542, 58543, 58544, 58548, 58550, 58552, 58553, 58554, 58570, 58571, 58572, 58573, 58578, 58951, 58953, 58956, 58575, 00846, 01962, 51925, G9774 | 68.31, 68.39, 68.41, 68.49, 68.51, 68.59, 68.9, 68.61, 68.69, 68.71, 68.79, 618.5, V88.01, V88.02 | 0UT44ZZ, 0UT90ZL, 0UT90ZZ, 0UT94ZL, 0UT94ZZ, 0UT97ZZ, 0UT9FZZ, 0UT98ZZ |
| **MRI ultrasound surgery** | 0071T, 0072T, C9734 | - | - |
| **Myomectomy** | 58146, 58145, 58561, 58545, 58546, 58140 | 68.29 | 0U590ZZ, 0U593ZZ, 0U594ZZ, 0U597ZZ, 0U598ZZ, 0UB90ZZ, 0UB93ZZ, 0UB94ZZ, 0UB97ZZ, 0UB98ZZ |
| **Oophorectomy** | 58720, 58940, 58943, 58950, 58951, 58952, 58953, 58954, 58956, 58957, 58958, 58575, 58661, 58920, 59120, 59121, 59150, 59151 | V45.77, 65.31, 65.39, 65.41, 65.49, 65.51, 65.52, 65.53, 65.54, 65.61, 65.62, 65.63, 65.64 | 0UB04ZZ, 0UB10ZZ, 0UB14ZZ, 0UB17ZZ, 0UB20ZZ, 0UB24ZZ, 0UT00ZZ, 0UT04ZZ, 0UT07ZZ, 0UT10ZZ, 0UT14ZZ, 0UT17ZZ, 0UT20ZZ, 0UT24ZZ, 0UT27ZZ |
| **Radiofrequency ablation** | 0404T, 58674, 0336T | - | - |
| **UAE** | 37243, 37204, 37210 | 68.24, 68.25, 38.80, 39.79, 99.29 | 04LE0DT, 04LE3DT, 04LE4DT, 04LF0DU, 04LF3DU, 04LF4DU, 04LE0CT, 04LE0ZT, 04LE3CT, 04LE3ZT, 04LE4CT, 04LE4ZT, 04LF0CU, 04LF0ZU, 04LF3CU, 04LF3ZU, 04LF4CU, 04LF4ZU |

Abbreviations: CPT = Current Procedural Terminology; HCPCS = Healthcare Common Procedure Coding System; ICD = International Classification of Diseases; MRI = magnetic resonance imaging; UAE = uterine artery embolization.

Supplemental **Table 3. Patterns of Use of Prescription Primary Hormonal Treatments Used During Follow-Up by Racial Groups**

| **Characteristic** | **Optum Commercial** | | **IBM Medicaid** | |
| --- | --- | --- | --- | --- |
|  | **White**  **(n=46,139)** | **Black**  **(n=17,297)** | **White**  **(n=7,353)** | **Black**  **(n=16,776)** |
| **Primary hormonal treatment received as initial post-index treatment** | 17,691 (38.3) | 6,112 (35.3) | 2,828 (38.5) | 7,118 (42.4) |
| Hormonal contraceptives | 11,350 (64.2) | 3,942 (64.5) | 1,632 (57.7) | 3,930 (55.2) |
| Hormonal contraceptives \| GnRH agonists | 16 (0.1) | 8 (0.1) | 12 (0.4) | 36 (0.5) |
| Hormonal contraceptives \| GnRH agonists \| Steroid hormones | - | - | 4 (0.1) | 16 (0.2) |
| Hormonal contraceptives \| GnRH antagonists | 2 (0.0) | 1 (0.0) | - | 1 (0.0) |
| Hormonal contraceptives \| Steroid hormones | 3,436 (19.4) | 1,622 (26.5) | 972 (34.4) | 2,706 (38.0) |
| GnRH agonists | 55 (0.3) | 43 (0.7) | 74 (2.6) | 270 (3.8) |
| GnRH antagonists | 58 (0.3) | 11 (0.2) | 2 (0.1) | 2 (0.0) |
| GnRH antagonists \| Steroid hormones | 14 (0.1) | 2 (0.0) | - | - |
| GnRH agonists \| GnRH antagonists | 3 (0.0) | 2 (0.0) | - | - |
| GnRH agonists \| Steroid hormones | 3 (0.0) | 2 (0.0) | - | - |
| Steroid hormones | 2,754 (15.6) | 478 (7.8) | 132 (4.7) | 157 (2.2) |
| **Primary hormonal treatment received anytime during follow-up** | 21,410 (46.4) | 7,395 (42.8) | 3,389 (46.1) | 8,910 (53.1) |
| Hormonal contraceptives | 12,350 (57.7) | 4,349 (58.8) | 1,810 (53.4) | 4,601 (51.6) |
| Hormonal contraceptives \| GnRH agonists | 41 (0.2) | 23 (0.3) | 43 (1.3) | 138 (1.5) |
| Hormonal contraceptives \| GnRH agonists \| Steroid hormones | - | - | 7 (0.2) | 38 (0.4) |
| Hormonal contraceptives \| GnRH antagonists | 11 (0.1) | - | - | 2 (0.0) |
| Hormonal contraceptives \| Steroid hormones | 4,514 (21.1) | 2,035 (27.5) | 1,191 (35.1) | 3,400 (38.2) |
| GnRH agonists | 263 (1.2) | 110 (1.5) | 127 (3.7) | 447 (5.0) |
| GnRH antagonists | 208 (1.0) | 58 (0.8) | 5 (0.1) | 9 (0.1) |
| GnRH antagonists \| Steroid hormones | 52 (0.2) | 15 (0.2) | 1 (0.0) | - |
| GnRH agonists \| GnRH antagonists | 14 (0.1) | 7 (0.1) | - | 2 (0.0) |
| GnRH agonists \| Steroid hormones | 24 (0.1) | 15 (0.2) | 2 (0.1) | 2 (0.0) |
| GnRH agonists \| GnRH antagonists \| Steroid hormones | - | 3 (0.0) | - | - |
| Steroid hormones | 3,923 (18.3) | 776 (10.5) | 203 (6.0) | 268 (3.0) |

Abbreviation: GnRH = gonadotropin-releasing hormone.

^a^ Numbers in groups are not mutually exclusive because the same patient could receive multiple types of hormonal therapies.

Supplemental **Figure 1. Study Schema**

**
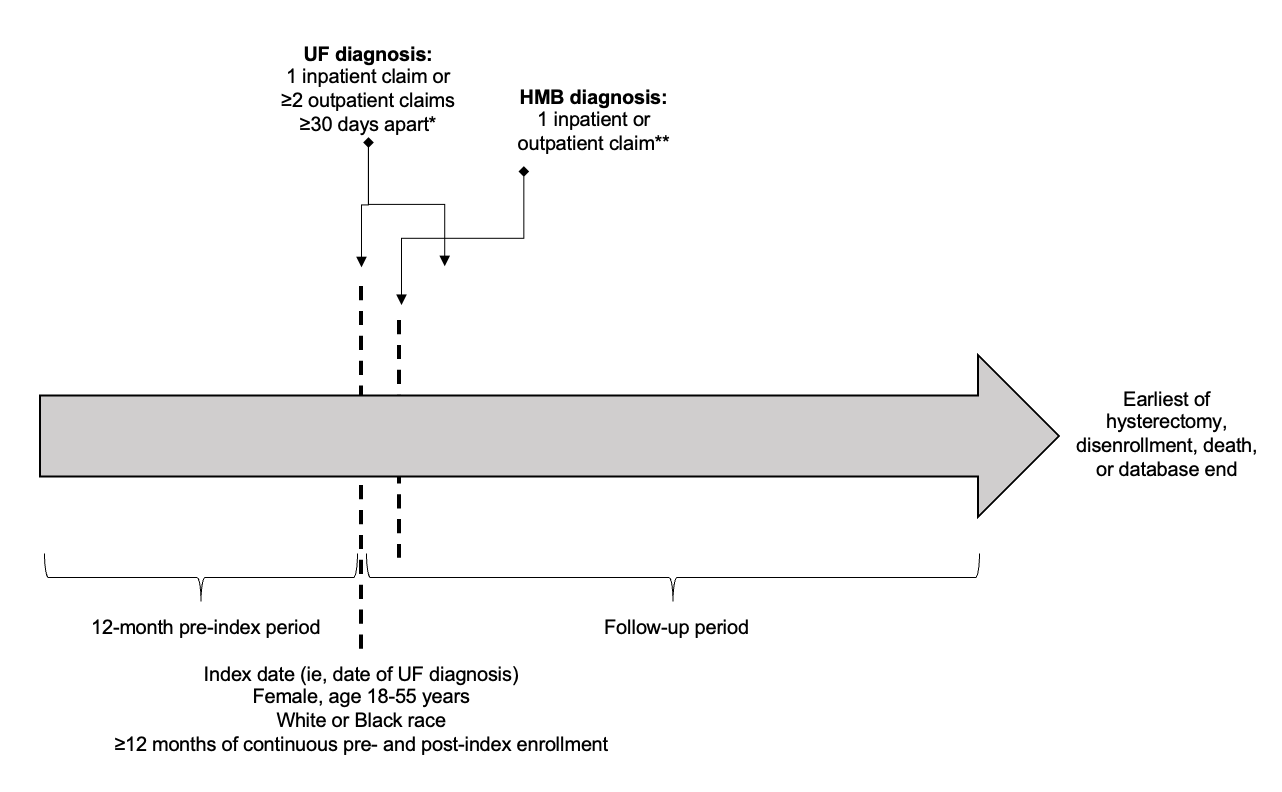
**

Abbreviations: HMB = heavy menstrual bleeding; ICD = International Classification of Diseases; UF = uterine fibroid.

*ICD-9 218.x or ICD-10 D25.x.

**The HMB diagnosis may have occurred before or after a UF diagnosis or on the same day (and/or claim) as a UF diagnosis. ICD-9 626.2, 626.4-626.6, 626.8, 626.9, 627.0 or ICD-10 N92.0, N92.1, N92.3-N92.6, N89.7, N93.1, N93.8, N93.9.

Supplemental **Figure 2. Patient Attrition**

**
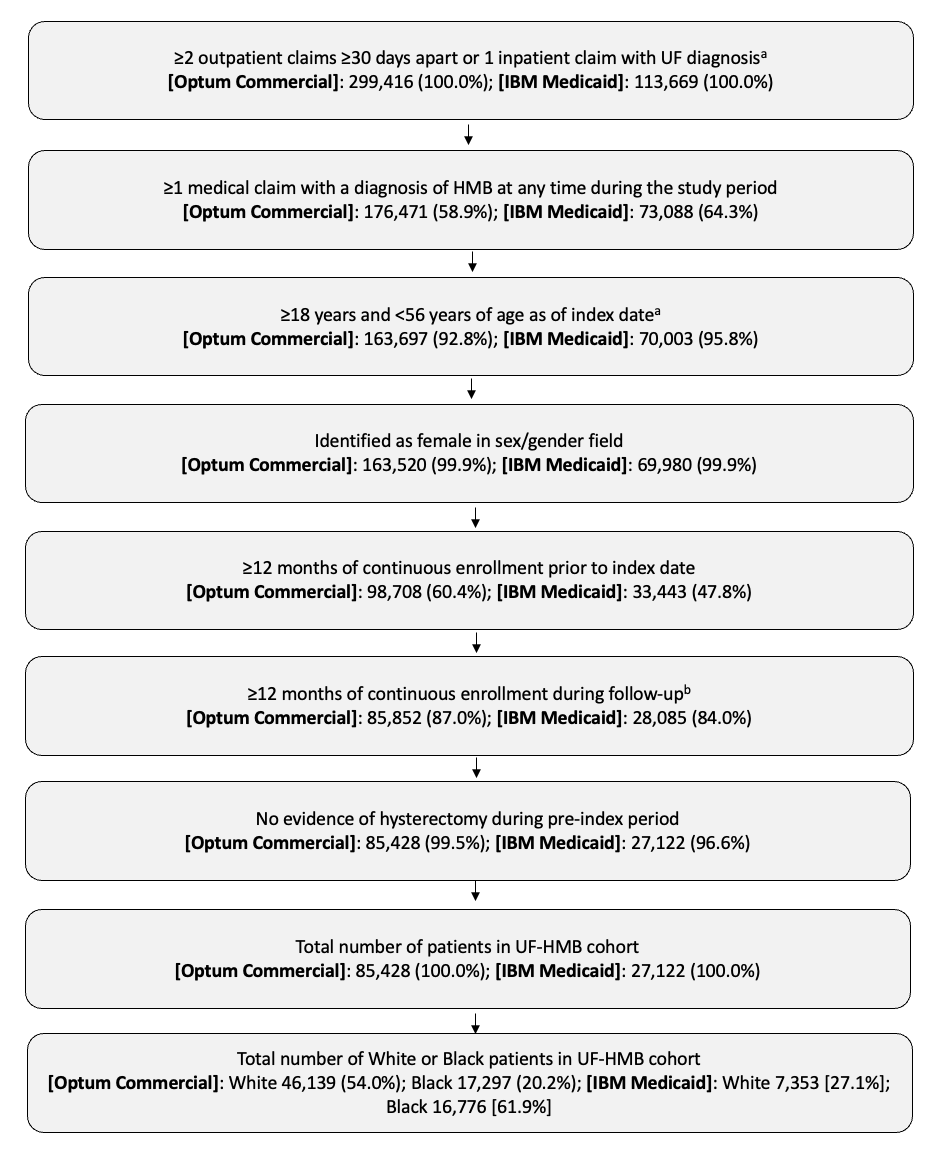
**

^a^ The index date was defined as the first claim corresponding to a UF diagnosis.

^b^ Or until the date of a hysterectomy (for patients who underwent this procedure <12 months following the index date).

Abbreviations: HMB = heavy menstrual bleeding; UF = uterine fibroids.

Supplemental **Figure 3. Percentage of Patients With Bulk Symptoms During Pre-Index by Race, (A) Optum Commercial and (B) IBM Medicaid Databases.**

*Denotes a statistically significant difference by race.

**
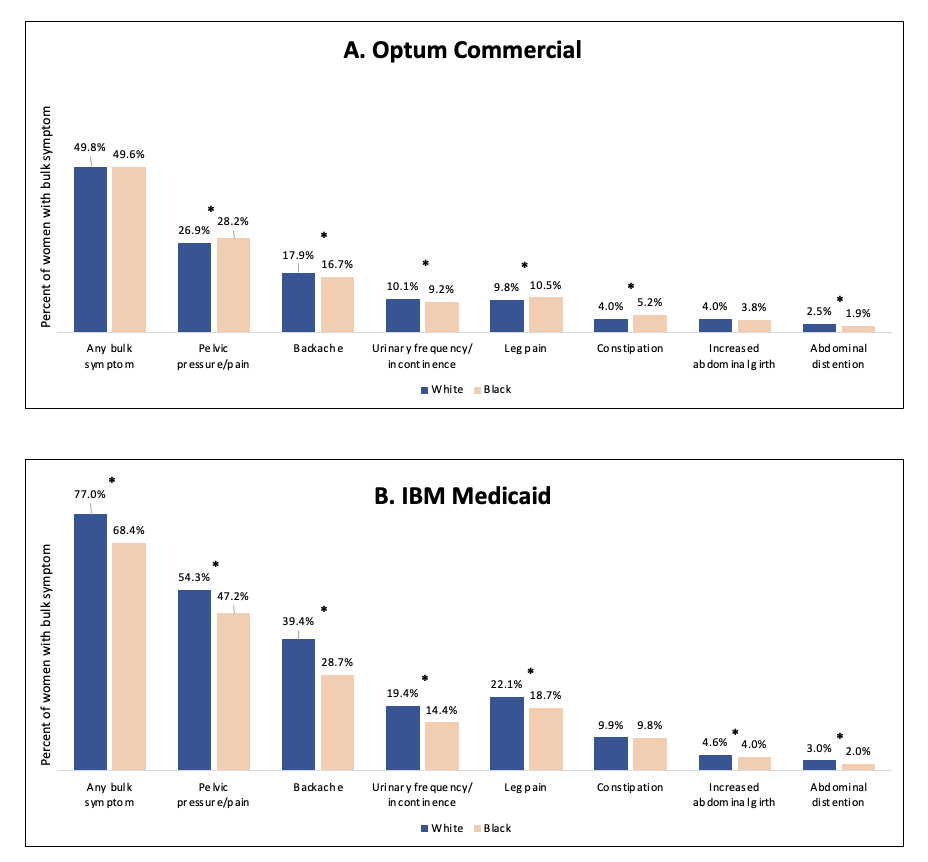
**
